# Research trends and hotspots in exercise interventions for liver cirrhosis: A bibliometric analysis via CiteSpace

**DOI:** 10.1097/MD.0000000000038831

**Published:** 2024-07-12

**Authors:** Tao Wei, Qiguan Jin

**Affiliations:** a Department of Physical Education, Yangzhou University, Yangzhou, Jiangsu, China.

**Keywords:** Cirrhosis, CiteSpace, exercise intervention, visualization analysis

## Abstract

Cirrhosis is a chronic liver disease with severe consequences for a patient’s health and survival. Exercise is an essential therapeutic strategy for both cirrhosis prevention and treatment. On the other hand, information regarding the present status of exercise-related research in cirrhosis is limited. Therefore, this study seeks to close the information gap in the scientific literature by using bibliometric techniques to analyze the trends, focal points, and cutting-edge research areas on exercise and cirrhosis. On September 22, 2023, research articles and reviews on exercise intervention for cirrhosis were obtained and downloaded from the Web of Science Core Collection (WoSCC). Subsequently, we employed CiteSpace (version 6.1.R6) to conduct bibliometric and knowledge graph analyses. 588 papers in 301 scholarly journals were written by 673 authors from 460 institutions spread over 63 countries and regions. The most productive nation among them is the United States. Not only is Zobair M. Younossi 1 of the most prolific writers, but he also receives the most co-citations. Most articles were published by the University of Michigan in the US, with the University of Alberta in Canada coming in second. Meanwhile, the WORLD JOURNAL OF GASTROENTEROLOGY has the most published articles, whereas HEPATOLOGY has the greatest number of co-citations. Apart from the theme words, the most frequently utilized keywords were “quality of life,” “insulin resistance,” and “mortality.” Future research may concentrate on “obesity,” “sarcopenia,” and “Mediterranean diet,” according to the analysis of keyword emergence. CiteSpace is used in this work to visually represent the topic of exercise intervention in cirrhosis, offering valuable information to researchers regarding the field’s current status and possible future direction.

## 1. Introduction

The hallmark of cirrhosis, a slowly developing and progressive liver disease, is the advanced replacement of typical liver tissue structure with scar tissue.^[1]^ A patient’s general health may suffer from various consequences from this pathological process, which causes structural abnormalities and reduced liver function. Several conditions, including cholestasis, drug or alcohol abuse, chronic hepatitis, and fatty liver, might contribute to the pathological process of cirrhosis.^[2–4]^ These elements cause the liver’s fibroblastic and inflammatory cells to react, which results in the deposition of the fibrous matrix, aberrant liver regeneration, and cell death. After the onset of cirrhosis, patients face the possibility of developing hepatocellular carcinoma (HCC) and experiencing liver dysfunction,^[5]^ which may manifest as portal hypertension, ascites, and hepatic encephalopathy.^[6,7]^ Taking into account the severe cirrhosis effects on the life quality of the patient and capacity for employment, there is a pressing necessity to investigate and create affordable and readily applicable methods of rehabilitation.

Exercise therapy, an emerging avenue that has received much attention, is widely regarded as an efficacious approach to both avert and manage liver cirrhosis. Exercise that is moderate in intensity lowers the burden on the liver,^[8]^ enhances hepatic fibrosis and inflammation, reduces the incidence of cardiovascular disease significantly,^[9]^ and enhances cardiovascular health in cirrhosis patients.^[10,11]^ Moreover, cirrhotic patients frequently exhibit decreased muscle mass and physical function, which is independently linked to morbidity and death both before and after liver transplantation.^[12,13]^ However, strength training or resistance exercises can increase muscle strength and endurance and improve cirrhotic patients’ capacities to perform daily life activities.^[14,15]^

The application of information visualization software in academic research is becoming increasingly popular. As 1 of the mainstream tools in the field of bibliometric research, CiteSpace software developed by Professor Chaomei Chen helps researchers to reveal the knowledge structure and research hotspots in the academic field by analyzing the citation relationships and textual content of scientific literature.^[16]^ Traditionally, bibliometric analyses have had many applications in medical research. For example, bibliometric analyses have been successfully applied to cancer research, cardiovascular disease research, and public health to help researchers understand the state of the art in these fields and identify new research directions. Bibliometric analysis can quantify a particular field’s development and knowledge structure and visualize the key elements and their interrelationships more intuitively. Although CiteSpace has achieved significant results in several areas, relatively few applications have been made in exercise intervention for cirrhosis. Current studies have focused on specific interventions and outcome assessments and need more systematic and global analyses, which has led to difficulties for researchers in understanding the trajectory of the field, its key contributors, and their research priorities. Therefore, this study used CiteSpace software to conduct visual analyses of countries, institutions, authors, and keywords to demonstrate the current status, hotspots, and future international research trends on exercise interventions for liver cirrhosis. Through these analyses, this study aims to provide academics with a systematic and comprehensive vision of exercise intervention in cirrhosis research, to provide references and insights for future research, and to promote scientific research and applied practice in this vital field.

## 2. Data and methods

### 2.1. Literature sources and search strategies

**Figure 1.**
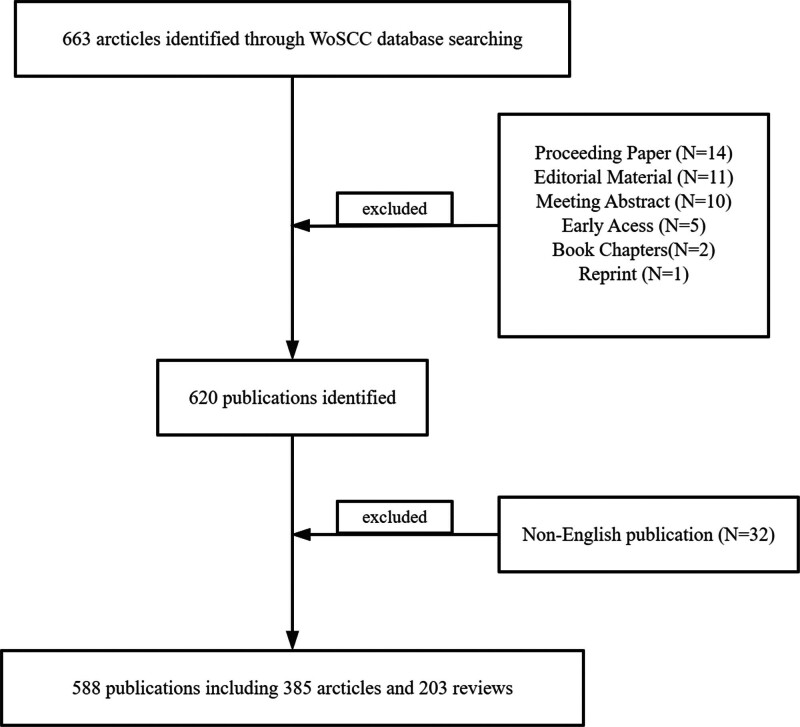
Flowchart for screening literature.

The authors performed a literature search on September 22, 2023, utilizing the Web of Science Core Collection (WoSCC) database. Web of Science is widely acknowledged by researchers as a database of excellent scientific resources.^[17]^ The following was the technique for searching the literature: (TS = (physical activity OR physical exercise OR exercise therapy) AND TS = (liver cirrhosis OR cirrhosis OR hepatic cirrhosis)), with the search language set to “English” and the item type limited to “Article” and “Review.” The range of dates was “all years.” A total of 588 publications on exercise and cirrhosis were identified. Figure 1 shows the publishing screening procedure flowchart.

### 2.1. Methods

588 documents were extracted and saved as “download_.txt” files in the plain text format of “Full Record and Cited References.” After that, CiteSpace 6.1.R6 software was used to load these files for additional analysis. CiteSpace built a visual knowledge network made up of lines and nodes. Nodes in distinct networks correspond to diverse research items, such as countries, institutions, authors, and keywords. Nodes’ sizes show how frequently they occur, while the color and thickness of their inner circles show how frequently they occur throughout different periods. The co-occurrence relationship is shown by the connecting lines that run between the nodes; the strength of the co-occurrence is indicated by the line’s thickness. The relationship between nodes is tighter, and the more and thicker the connections are. The centrality of a node means its value inside the network. A node characterized by a purple outer circle is regarded as a critical or turning point in CiteSpace,^[18]^ owing to its elevated intermediate centrality. Trend plots of annual publications and citations were produced using Microsoft Excel (2021).

The following parameters are configured: time slicing = 1999 to 2023; year perslice = 1; node types = country, author, cited author, cited reference, keywords; pruning = pathfinder and puning sliced networks; threshold selection criteria = the top 25 results; (6) top n = 50. The remaining values are set to default.

## 3. Results

### 3.1. Analysis of annual publications

**Figure 2.**
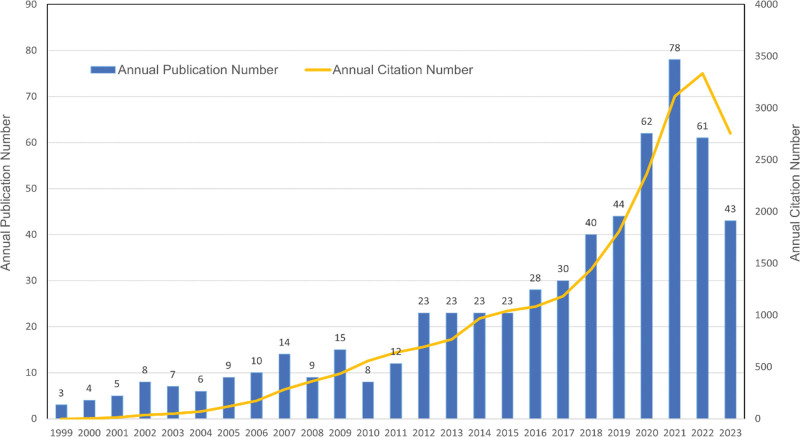
Global publication trends and total citations for exercise interventions in cirrhosis from 1999 to 2023.

A grand total of 588 research papers were published, comprising 385 articles (65.47%) and 203 reviews (34.52%). Academic research on exercise intervention for cirrhosis began to appear in 1999, as Figure 2 illustrates, the quantity of articles is steadily increasing on an annual basis. Although the analysis in this paper only covers those published between January and September, the number of articles published in 2023 is expected to continue to increase. There were just 110 paper publications between 1999 and 2011, at an average of 8.46 each year. These publications received 2745 citations in total, or 24.95 citations per piece. This phase, which is still in its early stages of exercise therapies for cirrhosis, has yet to get much attention. Nevertheless, there was a notable surge in published articles between 2012 and 2023, amounting to 478, with an annual average of 40.50 papers (a record of 11.8 years till September 2023). These articles received 20,561 citations in total, or 43.01 quotations on average. It means that researchers have increasingly focused on the significance of exercise in the treatment of cirrhosis, considering it to be a crucial area of research.

### 3.2. Analysis of countries/regions and institutions

**Figure 3.**
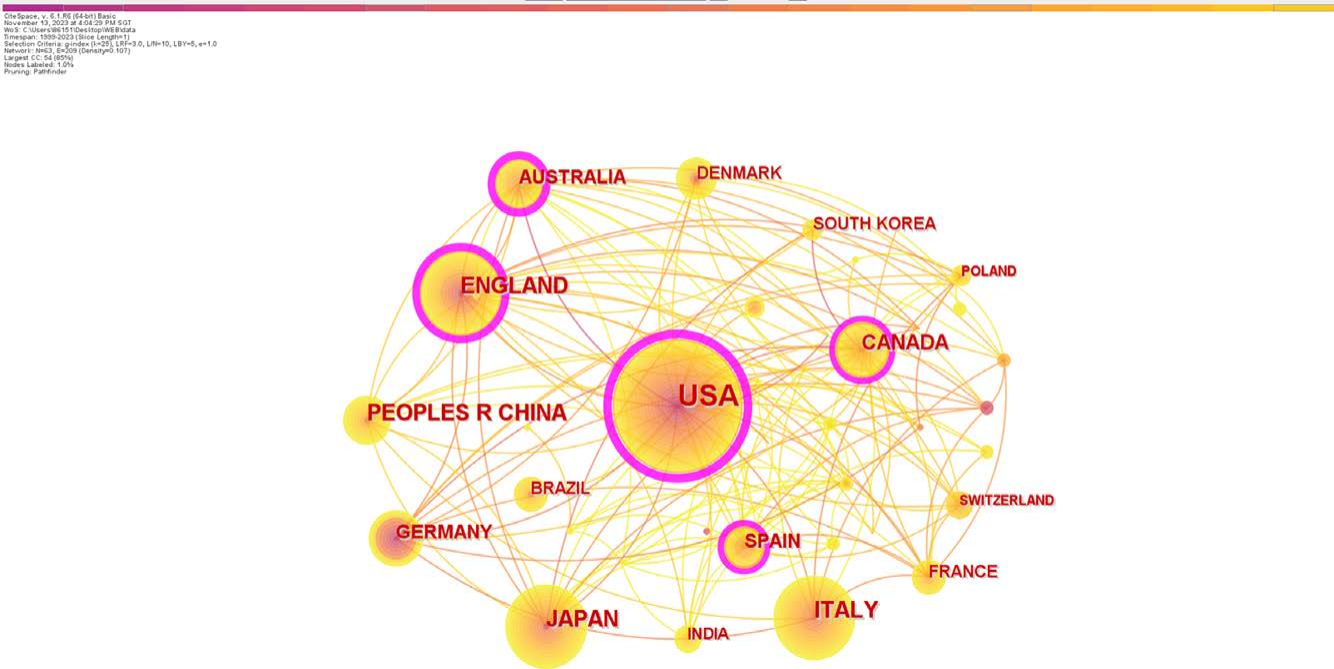
Collaboration network of countries or regions.

Mapping and analysis of country cooperation networks using “countries” as nodes (Fig. 3). According to the dataset, 64 countries/regions have published studies on exercise intervention for liver cirrhosis, mainly in the Americas, Europe, and Asia. According to the data in Table 1, the United States tops the list regarding the number of articles issued, accounting for approximately one-third (186) of the total literature collected, showing its activity in cirrhosis research. ENGLAND (57) and ITALY (51) ranked second and third, respectively. Canada, Australia, and the United States are the 3 countries/regions that rank highest in terms of citation frequency. Conversely, the nations with the most average citations per item are Canada, Australia, and Germany. The H-index, a composite quantitative index used to assess a researcher’s scholarly impact, was calculated using the highest value of H, i.e., H papers with at least H citations.^[19]^ As expected, the United States has the highest H-index at 48, much higher than ITALY (27) and ENGLAND (23). With the most documents, citation rates, H-index, and centrality, the United States leads the field in this area. While China and Japan have comparatively high numbers of publications, their academic influence is limited, and their citations are relatively low.

**Table 1:** Top 10 most prolific countries in the field of exercise intervention for cirrhosis.

Rank	Country/region	Publications	Citations	Average citations per item	Centrality	H-index
1	USA	186	9910	53.28	0.44	48
2	ENGLAND	57	2089	36.65	0.21	23
3	ITALY	51	2200	43.14	0.02	27
4	JAPAN	50	1520	30.4	0.05	18
5	PEOPLES R CHINA	48	1635	34.06	0.07	17
6	CANADA	37	2852	77.08	0.11	20
7	AUSTRALIA	27	4145	153.52	0.2	17
8	SPAIN	25	1093	43.72	0.2	15
9	GERMANY	24	2371	98.79	0.02	16
10	BRAZIL	21	313	14.9	0	9

**Figure 4.**
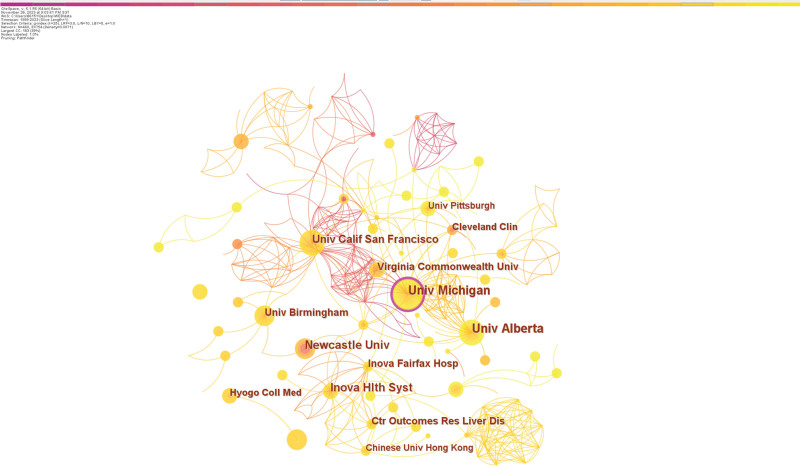
Collaboration network of organizations.

The top 5 colleges in terms of postings out of the 460 universities receiving the most attention are nearly all comprehensive universities: University of Michigan (20 articles), University of Alberta (17), Inova Health System (14), Newcastle University (13), and UC San Francisco (12). Only the University of Michigan (0.11) and Duke University (0.1) have centrality >0.1, as seen in Table 2. Despite few publications, Duke University has a high centrality score, indicating its unique place in exercise intervention research in cirrhosis. A comprehensive analysis of the number and centrality of article publications reveals that Michigan University and the University of Alberta are major research institutions in exercise interventions in cirrhosis. They both form the core of a complex network of collaborations. In this field, University of Michigan research has centered on the assessment of frailty indices in cirrhotic patients, nutritional and exercise interventions, and cirrhosis comorbid with sarcopenia, among others. Univ Alberta research focuses on the impact of multiple comorbidities on outcome measures after liver transplantation, the pathogenesis of cirrhosis, and the impact of exercise therapy on physical function, clinical symptoms, and biomarkers in patients with cirrhosis. Figure 4 illustrates the collaborative relationship between the different research organizations.

**Table 2:** Top 10 highly productive organizations in the field of exercise interventions for cirrhosis.

Rank	Institution	Publications	Rank	Institution	Centrality
1	Univ Michigan	20	1	Univ Michigan	0.11
2	Univ Alberta	17	2	Duke Univ	0.1
3	Inova Hlth Syst	14	3	Univ Alberta	0.09
4	Newcastle Univ	13	4	Inova Hlth Syst	0.08
5	Univ Calif San Francisco	12	5	Univ Pittsburgh	0.07
6	Inova Fairfax Hosp	10	6	Inst Salud Carlos III	0.07
7	Virginia Commonwealth Univ	10	7	Chinese Univ Hong Kong	0.06
8	Univ Birmingham	10	8	Inova Fairfax Hosp	0.05
9	Ctr Outcomes Res Liver Dis	9	9	Harvard Univ	0.05
10	Cleveland Clin	8	10	Univ Calif San Francisco	0.04

### 3.3. Analysis of authors and co-cited authors

**Figure 5.**
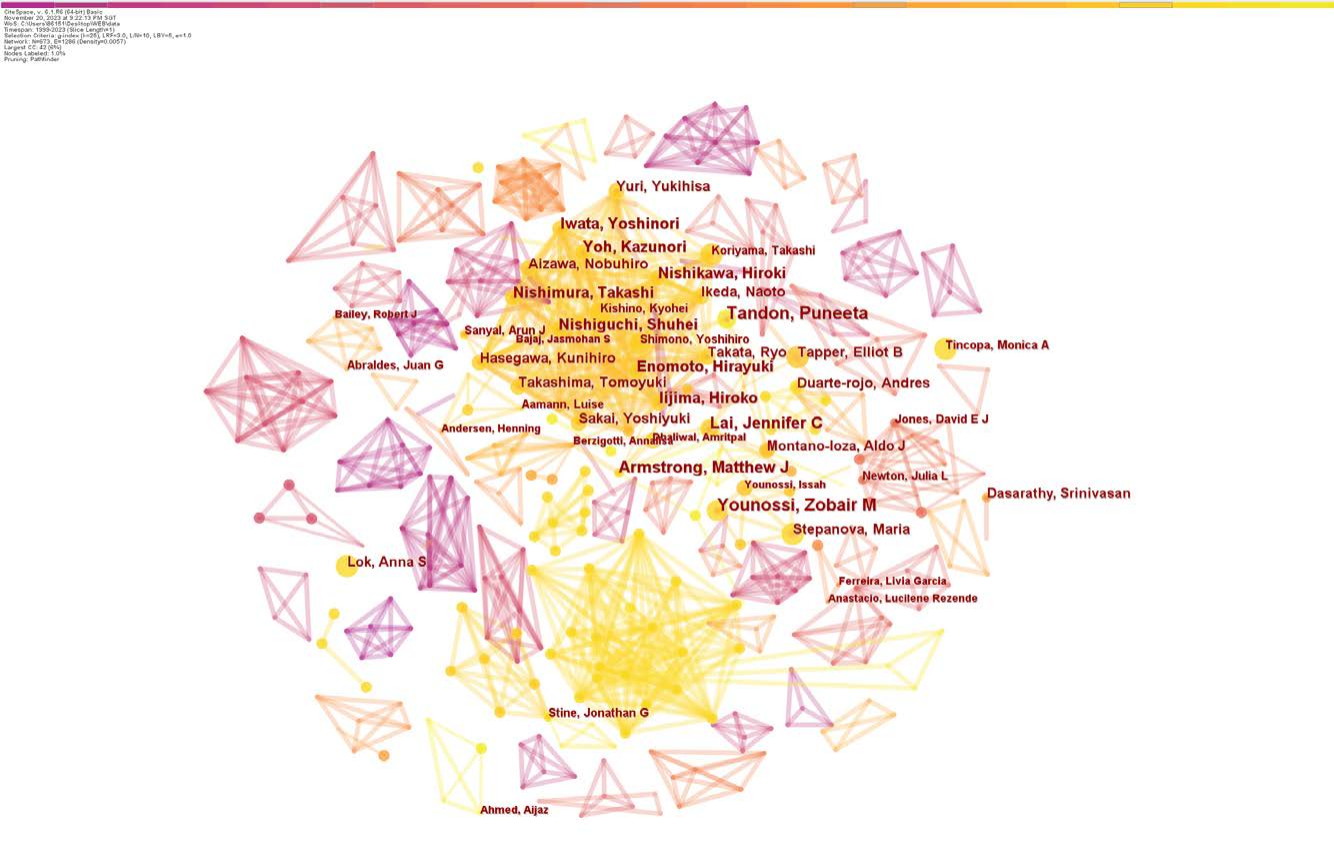
Collaboration network of authors.

The authors’ collaborative network mapping can reflect the collaborative relationship and tightness between different scholars in the field. 673 authors were involved in the publication of the 588 papers, and Table 3 presents the co-cited authors and the ten most prolific authors. Zobair M. Younossi, from the Department of Medicine at Inova Fairfax Medical Campus, authored 14 articles and received 688 citations. Puneeta from the University of Alberta’s Division of Gastroenterology came next with 14 papers and 676 citations. Third place went to Jennifer C. Lai of the University of California, San Francisco’s Department of Medicine, who had 11 articles and 676 citations. We also note that Dasarathy, Srinivasan (Case Comprehensive Cancer Center) was cited 1071 times and had the highest Average citations per item despite publishing only 10 articles. In addition, Zobair M. Younossi has the highest H-index. The other writers in the top 10 have between 9 and 10 papers published, with no discernible difference in that amount. As shown in Figure 5, exercise intervention in cirrhosis has developed many research teams, including many prolific authors. The primary research teams are Tandon P, Duarte-Rojo A, Amann L, Carey EJ, Bashir M, and Bemeur C.

**Table 3:** Top 10 most productive authors and co-cited authors in the field of exercise interventions for cirrhosis.

Rank	Author	Counts	Citations	Average citations per item	H-index	Co-cited author	Co-citations
1	Younossi, ZM	14	688	49.14	11	Younossi ZM	133
2	Tandon, P	14	676	48.29	8	Marchesini G	101
3	Lai, JC	11	676	61.45	9	Chalasani N	98
4	Armstrong, MJ	10	188	18.8	7	Sanyal AJ	89
5	Duarte-Rojo, A	10	480	48	7	Lai JC	85
6	Tapper, Elliot B	10	288	28.8	7	Tandon P	73
7	Stepanova, M	10	343	34.3	8	Montano-Loza AJ	73
8	Dasarathy, S	10	1071	107.1	8	Angulo P	72
9	Jones, DEJ	10	560	56	10	Ratziu V	70
10	Nishikawa, H	9	126	14	7	Carey EJ	64

**Figure 6.**
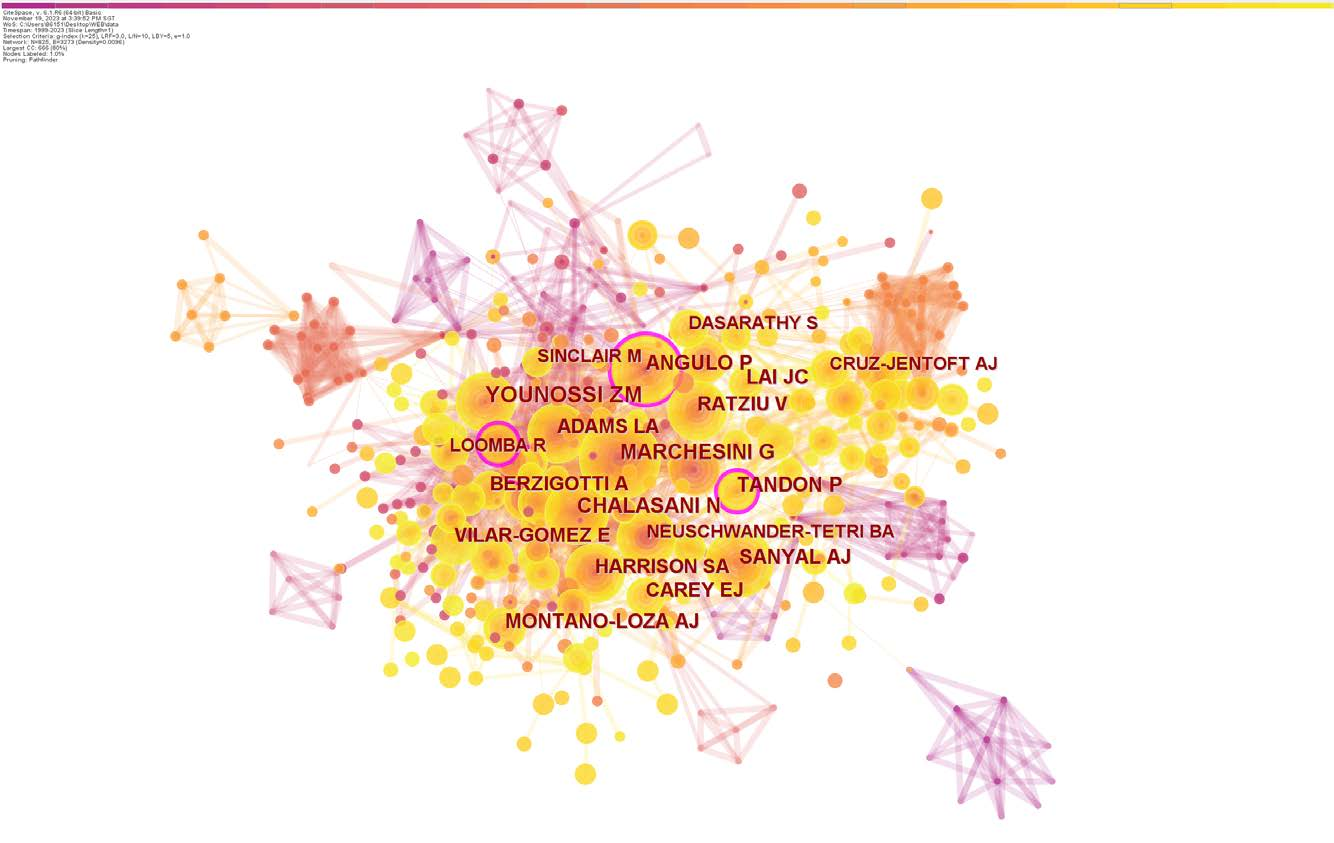
Collaboration network of cited authors.

As seen from the data in Table 3, Zobair M. Younossi (133 citations) is the author with the most co-citations and a prolific writer. This means that his paper has been widely cited in the work of other researchers, and his findings have been an essential contribution to developing the field of exercise intervention in cirrhosis. Sanyal AJ (89 citations), Chalasani N (98 citations), and Marchesini G (101 citations) come after him. A network map of co-cited writers is presented in Figure 6.

### 3.4. Analysis of journals and co-cited journals

According to the WoS database, 588 papers on exercise intervention in cirrhosis have been published in more than 300 academic journals. The ten highest-producing journals are shown in Table 4. The WORLD JOURNAL OF GASTROENTEROLOGY (4.3, Q2) accounted for 3.3% of the total number of publications, or 21 papers, the highest number of articles published. F Following this were 16 papers from HEPATOLOGY (13.5, Q1), 14 documents from DIGESTIVE DISEASES AND SCIENCES (3.1, Q3), 13 pieces from the journal of HEPATOLOGY (25.7, Q1), and 13 papers from NUTRIENTS (5.9, Q1). Interestingly, HEPATOLOGY has a high citation frequency of 3574, more than 3 times that of the WORLD JOURNAL OF GASTROENTEROLOGY, despite only having 16 papers published. It indicates that the academic articles published in HEPATOLOGY have vast influence and high theoretical value in this field. Out of the top 10 journals in terms of publication count, 5 journals are classified under JCR 1, and 6 journals have an impact factor (IF) over 5.

**Table 4:** Top 10 most productive journals and co-cited journals in the field of Exercise interventions for cirrhosis.

Rank	Journal	Counts	Citations	Average citations per item	IF and JCR (2022)	Co-cited journal	Co-citations	IF and JCR (2022)
1	WORLD JOURNAL OF GASTROENTEROLOGY	21	955	45.48	4.3, Q2	HEPATOLOGY	489	13.5, Q1
2	HEPATOLOGY	16	3581	223.81	13.5, Q1	J HEPATOL	435	25.7, Q1
3	DIGESTIVE DISEASES AND SCIENCES	14	331	23.64	3.1, Q3	GASTROENTEROLOGY	388	29.4, Q1
4	JOURNAL OF HEPATOLOGY	13	1219	93.77	25.7, Q1	CLIN GASTROENTEROL H	311	13.576, Q1
5	NUTRIENTS	13	376	28.92	5.9, Q1	AM J GASTROENTEROL	294	9.8, Q1
6	EUROPEAN JOURNAL OF GASTROENTEROLOGY & HEPATOLOGY	12	196	16.33	2.1, Q4	NEW ENGL J MED	254	176.082, Q1
7	LIVER TRANSPLANTION	12	477	39.74	4.6, Q2	WORLD JOURNAL OF GASTROENTEROLOGY	252	4.3, Q2
8	LIVER INTERNATIONAL	11	259	23.55	6.7, Q1	LIVER INT	249	6.7, Q1
9	JOURNAL OF GASTROENTEROLOGY AND HEPATOLOGY	10	294	29.4	4.1, Q2	GUT	241	31.793, Q1
10	AMERICAN JOURNAL OF GASTROENTEROLOGY	9	282	31.33	9.8, Q1	ALIMENT PHARM THER	237	9.524, Q1

**Figure 7.**
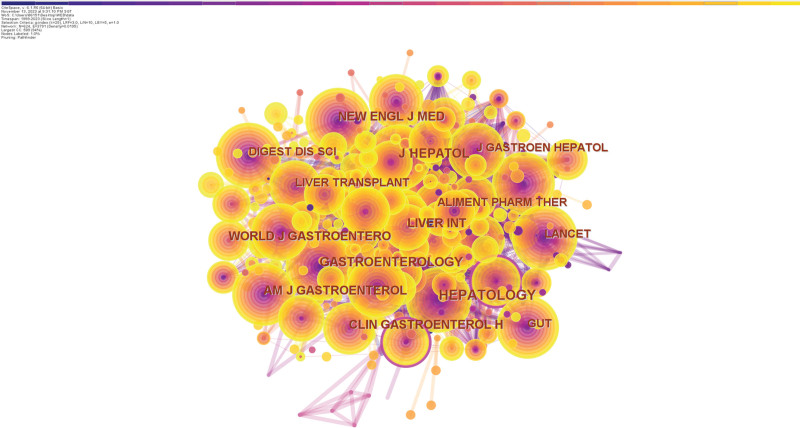
Network of cited journals.

An additional examination of co-cited journals was carried out to pinpoint important journals within the area. Table 4 and Figure 7 display the specific information on the co-cited journals. Out of 636 co-cited journals, a total of 5 journals had more than 300 co-citations. Of these, Among the journals in the field, HEPATOLOGY (13.5, Q1) Ranked highest in terms of co-citations, followed by J HEPATOL (25.7, Q1), GASTROENTEROLOGY (29.4, Q1), and CLIN GASTROENTEROL H (13.576, Q1). Of the ten journals with the highest total citations, 9 belong to the JCR 1 region, and 6 have an IF of more than 10.

### 3.5. Analysis of co-cited references

**Figure 8.**
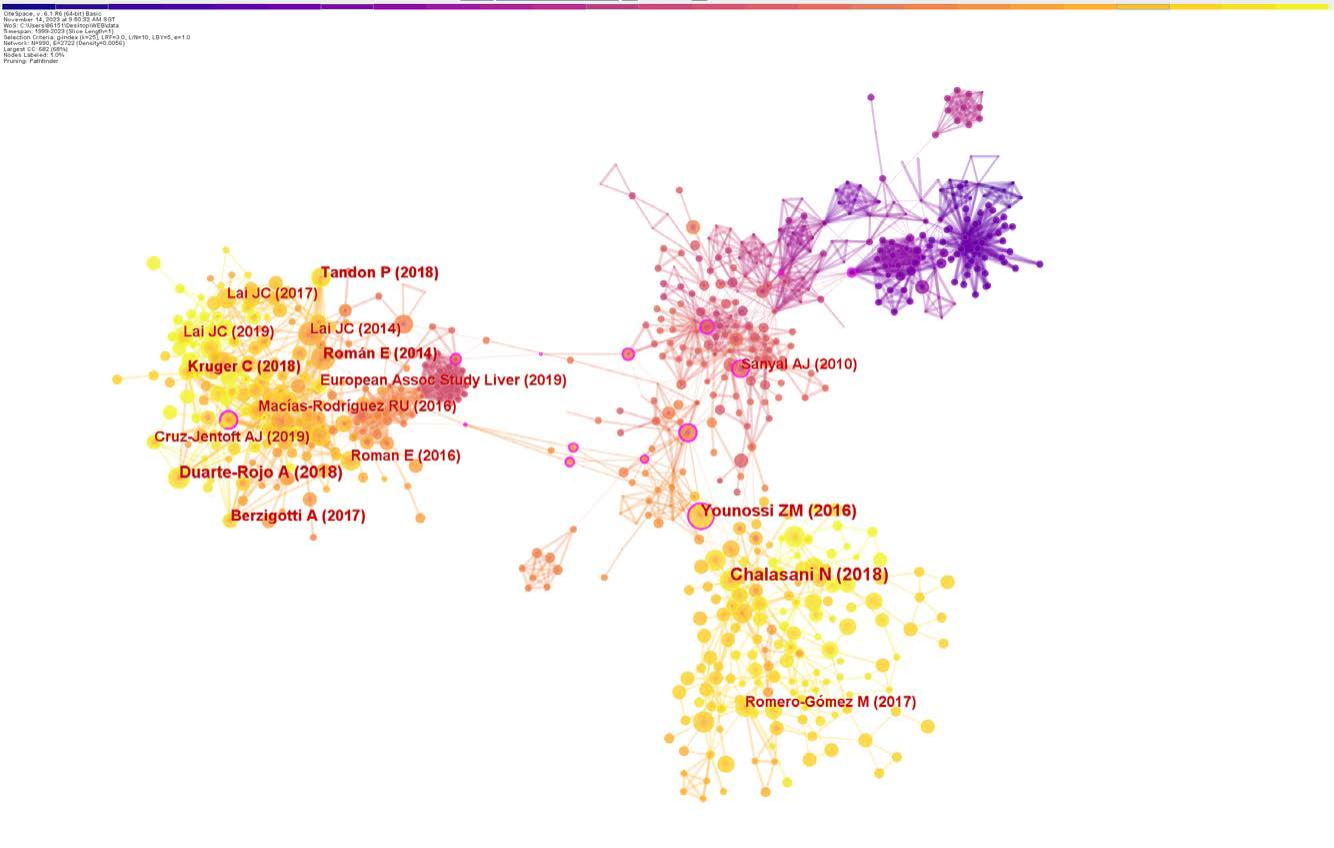
Documents co-citation network.

Co-cited references are co-citation relationships formed when 2 (or more) papers are simultaneously cited by 1 or more later articles, and literature with a higher co-citation frequency tends to reflect the knowledge base of a particular research area.^[20]^After launching CiteSpace, the node type was selected as “reference” without pruning the graph. Based on this strategy, we successfully constructed a co-cited literature atlas (Fig. 8). Table 5 lists the details of the 5 most frequently cited literature in exercise intervention for liver cirrhosis.

**Table 5:** Top 5 co-cited references in the field of exercise interventions for cirrhosis.

Co-citations	First author	Journal	Year	DOI	IF	Centrality
53	Chalasani N	HEPATOLOGY	2018	10.1002/hep.29367	13.5	0.04
39	Duarte-Rojo A	LIVER TRANSPLANT	2018	10.1002/lt.24958	4.6	0.02
37	Younossi ZM	HEPATOLOGY	2016	10.1002/hep.28431	13.5	0.17
30	Berzigotti A	HEPATOLOGY	2017	10.1002/hep.28992	13.5	0.03
30	Tandon P	J HEPATOL	2018	10.1016/j.jhep.2018.06.017	25.7	0.01

Firstly, in terms of content, the first literature was published by Chalasani N in 2018, which offers robust evidence-based backing for the issues related to the the identification, management, and avoidance of nonalcoholic fatty liver disease (NAFLD).^[21]^ This data informs further research, enhances the understanding of problems associated with NAFLD, and helps develop more effective solutions. The second highly referenced study looked at the benefits of exercise for individuals with advanced liver disease and cirrhosis, as well as the concomitant symptoms in these patients.^[22]^ It showed that exercise improves patients’ metabolic syndrome, sarcopenia, fitness level, and quality of life. However, supervised physical activity is impractical or unsustainable for the majority of patients. Therefore, the researchers propose an innovative home-based physical activity intervention to benefit more patients. The third study is a meta-analysis that thoroughly assesses the prevalence, incidence rate, progression, and outcomes of global non NAFLD.^[23]^ The study’s findings demonstrated that NAFLD presents a significantly high risk of metabolic complications and that many patients have underlying progressive liver disease, posing a significant public health challenge. The fourth highly cited literature is a research article by Berzigotti A published in 2017 in the journal HEPATOLOGY, which examined the effects of weight loss through lifestyle changes in overweight/obese cirrhotic patients.^[24]^ The study found that after a 16-week personalized low-calorie lifestyle intervention program, patients experienced significant weight loss and reduced portal vein pressure. The patient maintained her weight loss during the 6-month follow-up period; no clinical loss appeared noted. The fifth co-cited paper examined exercise’s advantages for cirrhosis patients.^[25]^ Exercise was found to dramatically improve muscle health, quality of life, fatigue, and hepatic venous pressure gradients in patients with cirrhosis without any side effects. In addition, the study provided information on nutrition and practical tips to promote physical activity in patients with cirrhosis.

**Figure 9.**
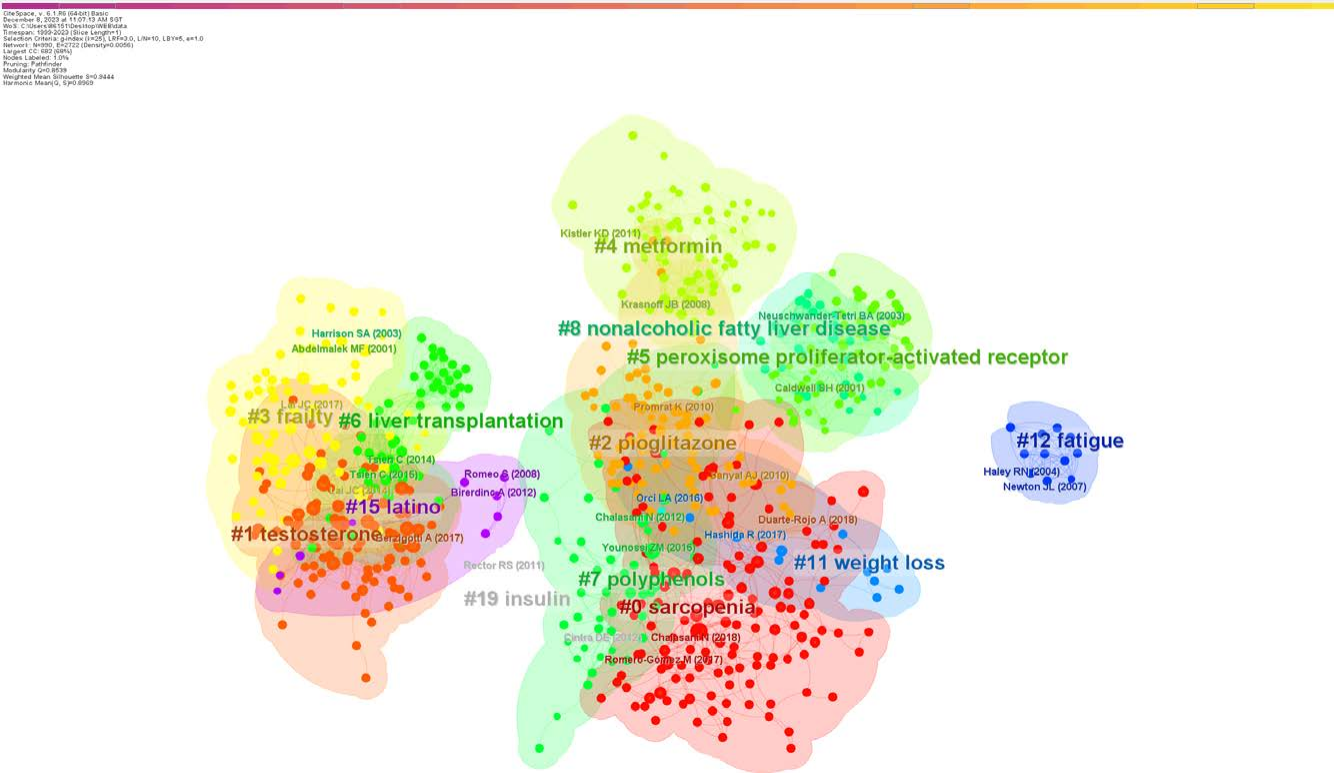
Documents co-citation network clustering diagram.

Next, we performed a network clustering analysis to clarify the themes of these cited documents. Cluster labels are noun phrases with significance extracted from keywords based on the logarithmic likelihood rate algorithm.^[26]^ Two metrics are provided by the CiteSpace software to evaluate the efficacy of mapping: the modularity (*Q* value) and the mean silhouette (*S* value), which are based on the network structure and clustering results.^[27]^ Generally, a *Q* value exceeding 0.3 signifies a significant clustering structure, whereas an *S* value exceeding 0.5 signifies a respectable clustering.^[28]^ In the clustering map we generated, the *Q* value is 0.8539, and the *S* value is 0.9444, indicating a significant and reliable clustering structure. Figure 9 shows the 9 clusters that contain the highest number of documents. The smaller the group tag sequence number, the more records the group contains. The 9 clustering clusters are: #0 sarcopenia; #1 testosterone; #2 pioglitazone; #3 frailty; #4 metformin; #5 peroxisome proliferator-activated receptor; #6 liver transplantation; #7 polyphenols and #8 nonalcoholic fatty liver disease.

**Figure 10.**
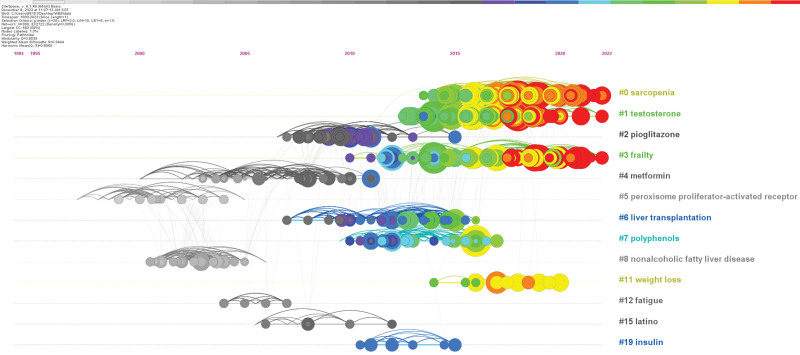
Documents co-citation network clustering timeline diagram.

We performed a timeline analysis of the co-cited literature to investigate the duration of each research hotspot and their interrelationships (Fig. 10). From the timeline, some research themes were initiated earlier, such as “peroxisome proliferator-activated receptor” and “metformin.” “Peroxisome proliferator-activated receptor” (PPAR) is a type of nuclear receptor linked with metabolic control that plays a critical regulatory role in the liver.^[29]^ Following particular research, PPAR activators may protect against cirrhosis by controlling hepatic lipid metabolism, lowering inflammatory and fibrotic reactions in the liver, and other processes.^[30]^ Another hot research topic is “metformin,” which is a commonly used oral hypoglycemic agent and is also widely used in diabetic patients. However, recent studies have revealed that metformin has some additional nonglycemic effects, which may positively affect cirrhosis by inhibiting fibrosis and attenuating inflammation.^[31,32]^ These findings suggest combining pharmacological and exercise interventions to treat cirrhosis. In recent years, “testosterone,” “frailty,” and “sarcopenia” have become the focus of research and remain active. Testosterone” is 1 of the leading sex hormones in men. In patients with cirrhosis, decreased testosterone levels are linked to higher morbidity and death rates.^[33]^ Appropriate exercise interventions may offer new therapeutic strategies for patients with cirrhosis by modulating testosterone levels. “Frailty” mainly refers to a state in which elderly or chronically ill patients are more likely to experience health problems in the face of external stressors. In this context, exercise interventions have positively alleviated frailty and improved patients’ overall health and quality of life. In addition, “sarcopenia” is a problem that often accompanies patients with cirrhosis, which results in a decrease in muscle function and a limitation of the patient’s mobility. It has been found that rational exercise training improves muscle mass and mobility in patients with cirrhosis and prevents further muscle damage. Based on these research hotspots, future cirrhosis treatment is expected to be more personalized. For example, the combination of medication during exercise intervention should focus on the individual’s sex hormone levels, frailty, and muscle health to develop a more precise and personalized treatment plan.

### 3.6. Keyword analysis

**Figure 11.**
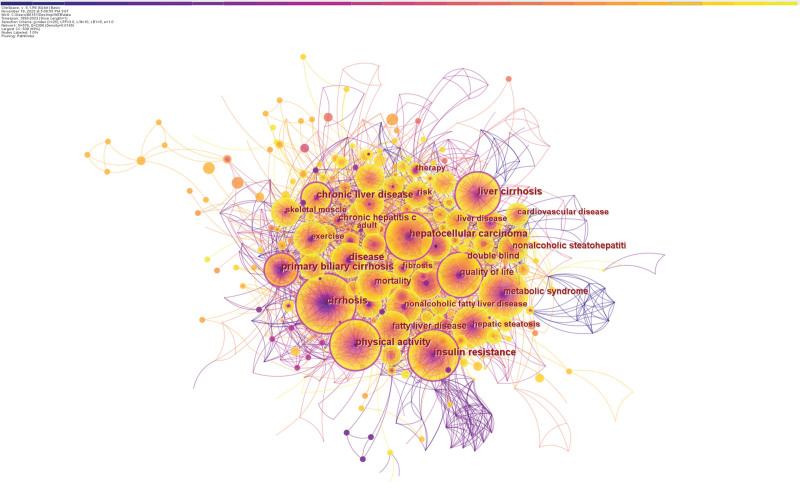
The network of keyword co-occurrences.

Keywords reflect the core content and ideas of the article. Through the analysis of keywords, we can provide a typical overview of research trends.^[34]^ We generated a keyword collinear network diagram with 576 nodes, 2396 network links, and a density of 0.0145. Figure 11 shows many keywords that are all related to each other. Due to limited space, Table 6 includes only the most often occurring 30 keywords due to space constraints. Exclusion of theme words such as “physical activity” and “Cirrhosis,” the top 5 high-frequency keywords are “quality of life,” “insulin resistance,” “hepatocellular carcinoma,” “ mortality,” and “metabolic syndrome.” In evaluating patients with liver cirrhosis, “quality of life” is an essential measure. It can objectively reflect patients’ overall well-being and satisfaction in physical, psychological, and social aspects and accurately reflect the impact of disease on their lives. Patients can enhance their physical and mental status through appropriate physical activities, improving their quality of life. The significance of this indicator suggests that researchers need to pay special attention to improving patients’ quality of life when designing exercise intervention programs. Besides, in patients with liver cirrhosis, there is a common phenomenon of “insulin resistance,” which is a common metabolic problem. Exercise has been shown to improve insulin sensitivity and slow the progression of cirrhosis by facilitating the process of glucose uptake and metabolism and reducing insulin resistance. “Hepatocellular carcinoma” and “mortality” are also hot topics in the field of cirrhosis research because cirrhosis is a significant risk factor for hepatocellular carcinoma and leads to a high death rate. Exercise interventions may be expected to reduce the risk of liver cancer incidence and mortality through various mechanisms, such as improving metabolic health and enhancing immune function. “Metabolic syndrome” is a combination of metabolic abnormalities caused by multiple factors, including obesity, hypertension, and abnormal lipid metabolism. These anomalies are strongly associated with the initial stages of liver cirrhosis and liver damage. The above high-frequency keywords suggest the need to incorporate exercise as a potential preventive measure in the long-term management of cirrhosis. Other high-frequency keywords include “hepatic steatosis,” “fatty liver disease,” “risk factor,” “weight loss,” and “sarcopenia.”

**Table 6:** Top 20 keywords in terms of frequency and centrality in the field of exercise interventions for cirrhosis.

Rank	Counts	Keywords	Centrality	Rank	Keywords	Counts	Centrality
1	140	Phys$ical activity	0.12	11	H$epatic steatosis	50	0.07
2	112	Cirrhosis	0.15	12	Fatty liver disease	50	0.04
3	90	Quality of life	0.13	13	Risk factor	44	0.04
4	86	Insulin resistance	0.11	14	Weight lose	43	0.02
5	71	Mortality	0.09	15	Sarcopenia	39	0.01
6	68	Hepatocellular carcinoma	0.12	16	Risk	38	0.05
7	63	Metabolic syndrome	0.08	17	Nonalcoholic steatohepatitis	37	0.05
8	63	Exercise	0.04	18	Chronic liver disease	36	0.11
9	59	Liver cirrhosis	0.14	19	Association	35	0.06
10	56	Disease	0.1	20	vitamin e	35	0.01

**Figure 12.**
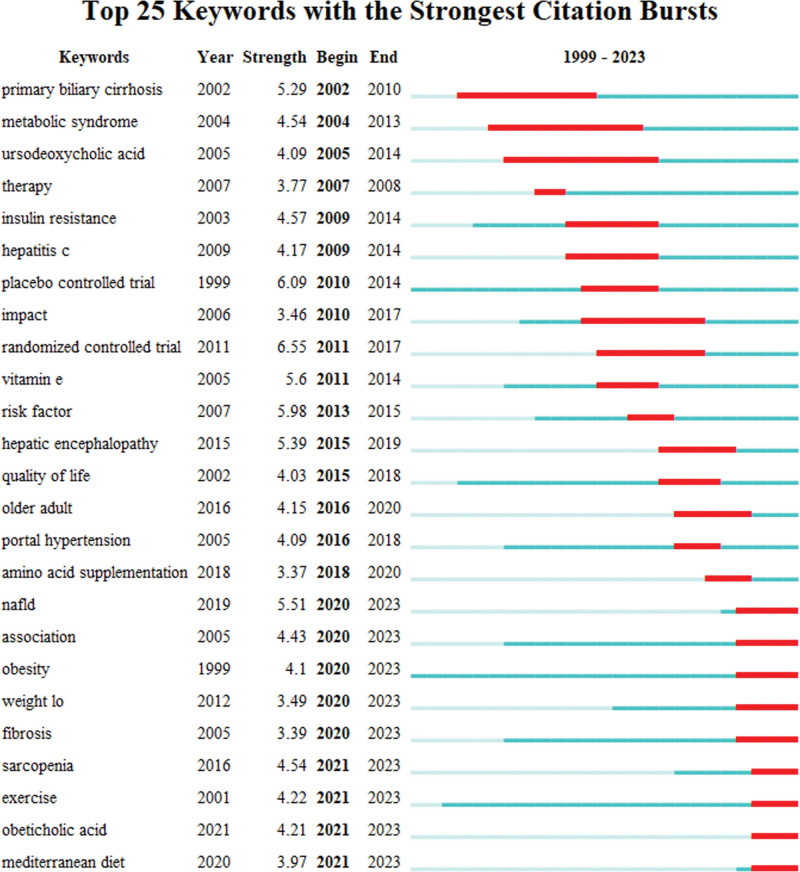
The keywords burstness analysis.

Keyword burst analysis can help researchers understand the hot topics, core concepts, and research trends in different time intervals in related fields more intuitively.^[27]^ The keyword burst analysis graph displays a red-colored segment to represent the period of keyword burst. The cyan solid line represents the presence of the keyword without any detected burst, while the cyan dashed line indicates that the keyword does not appear in this period. Because of this, we can assess the impact of the burst words by looking at their duration and burst intensity. A more extended continuity and a higher burst intensity rating indicate that the topic has had a more significant influence during that specific period. If a keyword has existed since its initial appearance, it can be regarded as a persistent area of study interest and a leading-edge subject. By analyzing the keywords through time series burst detection, we can get the top 25 keywords with the highest burst intensity (Fig. 12). Keywords of primary biliary cirrhosis, metabolic syndrome, ursodeoxycholic acid, insulin resistance, and hepatitis C suddenly increased from 2002 to 2014. This suggests that during this time, researchers have focused primarily on the etiology and pathomechanisms of cirrhosis and possible pharmacological treatments. From 2015 to 2019, hepatic encephalopathy, quality of life, and portal hypertension became the critical research areas. The focus during this period shifted significantly towards improving the quality of life and managing complications in patients with cirrhosis, suggesting a greater emphasis on these issues. In addition, key topics such as NAFLD, association, obesity, weight loss, fibrosis, sarcopenia, exercise, obeticholic acid, and the Mediterranean diet have received sustained attention recently. This change reflects the gradual expansion of research hotspots from etiology and pathological mechanisms to comprehensive management and preventive measures, primarily metabolic and lifestyle-related risk factors. Particularly noteworthy is that “randomized controlled trial” became the keyword with the highest outbreak intensity in 2013. It indicates that during this period, the scientific community began to adopt more rigorous study designs to assess the effects of different treatments and interventions. This enhanced the scientific validity and reliability of studies and provided an essential basis for confirming effective exercise interventions. Similarly, the explosion of the keyword “placebo-controlled trials” (strength 6.09) further confirms this trend.

## 4. Discussion

### 4.1. General Information

This study is the inaugural bibliometric analysis of exercise intervention for cirrhosis, utilizing the WoS database. As of September 22, 2023, 588 articles from 673 writers across 460 institutions in 63 nations and regions have been published in more than 300 journals. According to this study, scholarly investigations into exercise therapies for cirrhosis commenced in 1999, and the amount of academic literature on the subject has steadily increased each year. Especially from 2015, the number of articles on this topic increased rapidly, reaching 78 articles per year by 2021. The top 5 highly cited publications, as seen in Table 5, were all released within the previous ten years (2016–2019), suggesting that this period was high quality in developing the exercise and cirrhosis research field. Visual analyses by country/region showed that the United States is a world leader in this sector, ranking first in terms of number of publications (186), centrality (0.44), and H-index (48). This finding might be explained by the fact that the government has heavily funded scientific studies into cirrhosis because the disease has a comparatively high morbidity and mortality rate in the continental United States, burdening society and the economy accordingly. From an author contribution and co-authorship perspective, there are many international researchers in exercise rehabilitation for cirrhosis, but the research is relatively fragmented. Tandon, Puneeta, University of Alberta, Canada (14 articles, 676 citations, H-index 8) and Zobair M. Youno si, Inova Healthcare System, USA (14 articles, 688 citations, H-index 11) having the highest number of published works. Regarding institutional contributions, the University of Michigan and Alberta are the core institutions in the study of exercise interventions in cirrhosis, and their findings have been an essential guide in the field. WORLD JOURNAL OF GASTROENTEROLOGY and HEPATOLOGY were the journals with the most published articles and the most influential quantity of co-citations, respectively. In that order, the top 5 high-frequency and high-centrality keywords were physical activity, cirrhosis, insulin resistance, quality of life, and mortality. The keyword “randomized controlled trial” had the highest burst intensity, while keywords such as “sarcopenia” and “obesity” are emerging as research foci.

### 4.2. Research Hotspots

Keyword co-occurrence networks may indicate research hotspots and trends in a specific topic.^[35]^ Through the analysis of keyword frequency and centrality and other related indicators, we summarized the following research hotspots in the field of exercise intervention for liver cirrhosis:

Insulin resistance: A high occurrence of glucose metabolism disorders is observed in individuals with cirrhosis, with 60% to 80% of them experiencing impaired glucose tolerance (IGT) and around 10% to 15% eventually acquiring diabetes mellitus.^[36,37]^ Related to cirrhosis, IGT is primarily characterized by elevated blood insulin levels and reduced insulin sensitivity in the peripheral tissues.^[37,38]^ Reduced insulin secretion may have a detrimental effect on the course of the disease and the likelihood that a patient with chronic liver disease (CLD) will survive.^[39]^ Long-term progression of CLD leads to structural damage to liver tissue, which in turn affects hepatocyte function and the ability to maintain glucose homeostasis.^[40]^ Insulin resistance leads to increased lipolysis in adipose tissue, elevating free fatty acid levels in the blood, eventually entering the liver and accumulating fat.^[41]^ Furthermore, the buildup of specific lipids in the liver might result in compromised insulin signaling, impacting the liver’s ability to remove insulin.^[42]^ Therefore, proactive measures to prevent insulin resistance and timely correction of glucose metabolism abnormalities are vital to monitor disease progression and improve prognosis in cirrhotic patients. Physical exercise is thought to improve insulin sensitivity and increase the efficiency of insulin signaling through multiple mechanisms.^[43]^ In a randomized controlled trial, exercise training lasting 12 weeks significantly reduced liver fat in older obese patients and improved considerably systemic insulin resistance.^[44]^

Quality of life: Cirrhosis is a persistent liver illness that arises from various reasons, including chronic hepatitis, alcoholism, and hepatovirus infections. The condition causes long-term damage to the structure and function of the liver, adversely affecting normal metabolism and detoxification functions and the patient’s overall health. Patients with cirrhosis often experience uncomfortable symptoms such as fatigue, loss of appetite, dyspepsia, pain in the hepatic region, and loss of physical strength, which cause great inconvenience and distress in their daily lives.^[45]^ Furthermore, cirrhosis can lead to several problems, such as ascites, hepatocellular carcinoma, and hepatic encephalopathy, which can significantly worsen a patient’s quality of life. Patients with cirrhosis require frequent medical visits, have a long and exhausting treatment process, and have a heavy financial burden, which affects the fulfillment of family and social roles and challenges their quality of life. Research has indicated a strong correlation between the enhanced quality of life and the increased survival rates of those diagnosed with cirrhosis.^[46]^ In addition to conventional drug therapy, exercise training is an effective adjunctive therapy. Numerous reliable and consistent evidence suggests that regular participation in physical activity has multiple benefits for patients with cirrhosis, which include reduction of liver fat accumulation, improvement of physical function and psychological well-being, enhancement of immune function, and health-related quality of life.^[47,48]^ However, it is integral to note that sensible exercise training for patients with cirrhosis should be quantified and personalized individually.

Hepatocellular carcinoma: HCC is the predominant histological subtype of liver cancer and a significant contributor to cancer-related mortality globally.^[49]^ The great majority of hepatocellular carcinoma patients have underlying liver diseases, with cirrhosis being the most prevalent.^[50]^ Interestingly, however, only 2% to 5% of patients with cirrhosis eventually develop HCC.^[51]^ The development of HCC is usually the result of long-term asymptomatic CLD, the incidence of which is closely related to the major causes of cirrhosis (e.g., chronic viral hepatitis, excessive alcohol consumption, and intoxication).^[50,52]^ Although most patients with cirrhosis do not progress to HCC, the risk of developing HCC on top of cirrhosis remains. Therefore, to enhance survival and delay the evolution of hepatocellular carcinoma, patients with cirrhosis should have regular monitoring and an early diagnosis. Although exercise itself cannot directly prevent the passage of cirrhosis to HCC, it continues to have a crucial function in both the avoidance and management of cirrhosis and HCC. A recent meta-analysis of research revealed an inverse relationship between levels of physical activity and the incidence of HCC; the more you exercise, the lower your chances of developing HCC.^[53]^ Animal investigations have also verified the advantages of consistent physical activity in the progression of HCC.^[54]^ Nevertheless, additional research is required to examine the precise processes that connect exercise to the risk of HCC. To sum up, physical activity is essential to good health and may also lower the chance of tumor development.

### 4.3. Research frontiers

Keyword bursts show the cutting edge of study in a certain area at a certain time.^[55]^ The keywords “obesity,” “fibrosis,” and “sarcopenia” have been exploding since 2020 and continue to do so today, highlighting the latest cutting-edge topics in sports and cirrhosis research.

Sarcopenia: Data show that 30% to 70% of patients with cirrhosis suffer from sarcopenia, a condition associated with aging that is defined by a gradual decline in both skeletal muscle mass and muscle strength.^[56]^ This condition can result in physical impairment, impacting the patient’s quality of life and causing prolonged periods of inactivity. Lifestyle changes and reduced energy expenditure not only promote an increase in adipose tissue but also lead to a decrease in muscle mass and function. Interestingly, there may be an interaction between sarcopenia and insulin resistance. Since skeletal muscle is influential in insulin-mediated glucose metabolism,^[57]^ developing sarcopenia may induce insulin resistance. Furthermore, sarcopenia has been proven to behave as a separate prognostic factor in cirrhosis patients, indicating poor clinical outcomes such as reduced functional capacity, elevated incidence of clinically significant infections, and elevated death.^[58]^ Sarcopenia often continues after liver transplantation, possibly due to impaired skeletal muscle growth and protein accumulation resulting from lack of adequate physical activity or prolonged use of immunosuppressive drugs. The studies above show that skeletal muscle is crucial in the etiology of cirrhosis and is likely to benefit cirrhotic patients through treatment measures such as increasing muscle mass. Studies have shown that regular physical activity effectively prevents or ameliorates sarcopenia and its associated complications.^[59]^ In patients with cirrhosis, twelve weeks of progressive resistance training substantially increases quadriceps cross-sectional area and muscle strength.^[15]^ Similarly, resistance exercise enhances skeletal muscle mammalian target of rapamycin (mTOR) signaling, thereby promoting muscle anabolism.^[60]^

Obesity: Obesity, as a global disease, has been increasing in the prevalence of cirrhotic patients.^[61]^ Research has indicated that obesity can serve as a catalyst for the advancement of CLD and can also exacerbate the prognosis for those with cirrhosis.^[62]^ Patients undergoing liver transplantation who have a body mass index exceeding 35 kg/m2 are more likely to experience postoperative problems and death.^[63]^ A chronic and persistent state of low-grade systemic inflammation is caused by obesity, probably as a result of M1-type macrophages in adipose tissue being activated and release of pro-inflammatory substances such TNF-α, IL-6, and IL-8. The production of these inflammatory factors may decrease hepatic sensitivity to insulin, leading to insulin resistance. With the increasing problem of obesity, the percentage of patients requiring liver transplantation with comorbid obesity is also on the rise.^[64]^ Cirrhotic individuals with concomitant obesity frequently have decreased skeletal muscle mass and increased adipose tissue, which predisposes them to sarcopenic obesity, which increases the risk of physical impairment and disability.^[65]^ Several studies indicate that obesity can independently increase the risk of fibrosis development and cirrhosis of the live.^[66]^ Specifically, 43% of obese patients develop cirrhotic compensations such as ascites, encephalopathy, or jaundice. In contrast, only 14% of people with average weight and 31% of patients who are overweight experience these symptoms. In addition, the negative impact of obesity on portal hypertension should not be overlooked. A year-long pharmacological treatment was able to reduce portal pressure in normal-weight or overweight patients, whereas in obese patients, portal hypertension was significantly higher.^[67]^ It means that obesity status increases the risk of portal hypertension and that weight reduction and obesity control are essential for the prevention and management of portal hypertension. Exercise as an intervention to prevent and treat obesity is widely accepted and continues to be supported by academic research. A 16-week lifestyle intervention, including of a customized low-calorie diet and 60 minutes of supervised physical exercise weekly, has demonstrated efficacy in reducing body weight and portal vein pressure among patients who are overweight or obese.^[24]^

Fibrosis: Fibrosis is not a disease but an abnormal response that arises from tissue repair reactions following a wide range of tissue/organ injuries, especially during chronic disease-based inflammation.^[68]^ In the liver, prolonged inflammation and damage trigger a fibrotic response, leading to liver structure and function abnormalities. Hepatic fibrosis is a prevalent pathological phenomenon observed in all types of chronic liver disorders, and prolonged hepatic fibrosis may further progress to cirrhosis.^[69]^ Meanwhile, cirrhosis, the result of progressive fibrosis, has a poor prognosis and a high mortality rate.^[70]^ Consequently, timely intervention and control of fibrosis development are paramount to preventing or reducing the growth of cirrhosis. Multiple studies showed that engaging in physical activity has a beneficial impact on decreasing fibrosis levels. A cross-sectional study found that people who did more than twice the recommended level of weekly physical activity in the Leisure Time Physical Activity Guidelines reported a 59% decreased chance of liver fibrosis and a 63% lower threat of developing cirrhosis.^[11]^ Besides, 6 months of exercise training significantly improved the fibrosis status as well as the inflammatory response of the liver in individuals with metabolic syndrome.^[71]^ These findings further highlight the importance of physical activity as a nonpharmacological intervention to prevent and mitigate liver fibrosis and cirrhosis.

### 4.4. Limitations

Several limitations apply to this study. Firstly, the study searched a limited number of databases, and some literature needed to be included in the analyses, thus failing to comprehensively cover the latest research findings. Second, the study was limited to English literature, which may have some language bias. Therefore, future studies should broaden the scope of data retrieval and innovate relevant analysis methods to ensure the quality of literature data to present the research progress in this field more comprehensively and accurately.

## 5. Conclusion

This paper examines trends in cirrhosis exercise interventions and the frontiers of research in this area during the last 20 years, giving a foundation for establishing or upgrading exercise intervention strategies for cirrhotic patients. Researchers and clinicians have acknowledged mainly the significance of exercise therapies for cirrhosis. The results of this study will help promote collaboration between research teams and advance the use of exercise therapy in the clinical management of cirrhosis. Despite the limitations of this study, it provides valuable references and guidance for further exploration of hotspots, priorities, and trends in the field. Future studies should analyze the precise mechanism by which exercise ameliorates hepatic cirrhosis based on this study and further explore the impact of physical activity on hepatic function, metabolic levels, and the immune system, as well as the effects of the combined application of training and other therapeutic modalities. Furthermore, researchers could explore the applicability of different types and intensities of exercise to treating liver cirrhosis. Developing optimal exercise regimens and rehabilitation programs can maximize exercise interventions’ efficacy to enhance cirrhosis patients’ health status.

## Author contributions

**Conceptualization:** Tao Wei.

**Data curation:** Tao Wei.

**Software:** Tao Wei.

**Validation:** Tao Wei.

**Visualization:** Tao Wei.

**Writing – original draft:** Tao Wei.

**Writing – review & editing:** Tao Wei, Qiguan Jin.

**Supervision:** Qiguan Jin.
